# Supplementary material for: Distributed dynamic event-triggered algorithm for optimization problem with time delay
Source: PLoS One. 2024 Apr 18;19(4):e0299535. doi: 10.1371/journal.pone.0299535 (PMC11025798; doi:10.1371/journal.pone.0299535)
Supplement: Attachment — Submitted filename: Response to Reviewers.docx [file pone.0299535.s001.docx]

**Responses to Editors and Reviewers**

**“Distributed dynamic event-triggered algorithm for optimization problem with time delay”**

**Lu Jiang^1^, Lunchao Xia^1*^, Zhongyuan Zhao^1*^**

Firstly, we sincerely thank the editor for giving us this valuable opportunity to revise our paper. Additionally, we are very grateful for the helpful comments provided by the reviewers on our paper. We have fully embraced their suggestions and made thorough revisions to the paper. To facilitate the reading for the editor and reviewers, we have used red font in the revised paper to highlight the corresponding changes. Below is our response to all specific issues:

**Response to Academic Editor**

**Comments to the Author:** Thank you for submitting your manuscript to PLOS ONE. After careful consideration, we feel that it has merit but does not fully meet PLOS ONE’s publication criteria as it currently stands. Therefore, we invite you to submit a revised version of the manuscript that addresses the points raised during the review process. When submitting your revision, we need you to address these additional requirements.

**Reply:** Thank you so much for your diligent efforts throughout the review process. We greatly appreciate the recognition given to our paper by all the reviewers. We have carefully studied the relevant comments and made revisions accordingly.

1.Please ensure that your manuscript meets PLOS ONE's style requirements, including those for file naming.

**Reply:** We have adjusted the manuscript according to the template requirements, and the naming of the documents also meets the requirements

2.Thank you for stating the following in the Acknowledgments Section of your manuscript:"This research was supported by the National Natural Science Foundation of China (No. 246U23B2061, 62033010), the Natural Science Foundation of Jiangsu Province, China (No. 247BK20200824) and Postgraduate Research and Practice Innovation Program of Jiangsu 248 Province, China (No. SJCX23 0391)." We note that you have provided additional information within the Acknowledgements Section that is not currently declared in your Funding Statement. Please note that funding information should not appear in the Acknowledgments section or other areas of your manuscript. We will only publish funding information present in the Funding Statement section of the online submission form. Please remove any funding-related text from the manuscript and let us know how you would like to update your Funding Statement. Currently, your Funding Statement reads as follows:“This research was supported by the National Natural Science Foundation of China (No. 246U23B2061, 62033010), the Natural Science Foundation of Jiangsu Province, China (No. 247BK20200824) and Postgraduate Research and Practice Innovation Program of Jiangsu 248 Province, China (No. SJCX23 0391).” Please include your amended statements within your cover letter; we will change the online submission form on your behalf.

**Reply:** We have included our amended statements within our cover letter.

3.Thank you for stating the following financial disclosure :"This research was supported by the National Natural Science Foundation of China (No. 246U23B2061, 62033010), the Natural Science Foundation of Jiangsu Province, China (No. 247BK20200824) and Postgraduate Research and Practice Innovation Program of Jiangsu 248 Province, China (No. SJCX23 0391)." Please state what role the funders took in the study.  If the funders had no role, please state: "The funders had no role in study design, data collection and analysis, decision to publish, or preparation of the manuscript." If this statement is not correct you must amend it as needed. Please include this amended Role of Funder statement in your cover letter; we will change the online submission form on your behalf.

**Reply:** We have included the roles of the funders took in the study within our cover letter.

4.When completing the data availability statement of the submission form, you indicated that you will make your data available on acceptance. We strongly recommend all authors decide on a data sharing plan before acceptance, as the process can be lengthy and hold up publication timelines. Please note that, though access restrictions are acceptable now, your entire data will need to be made freely accessible if your manuscript is accepted for publication. This policy applies to all data except where public deposition would breach compliance with the protocol approved by your research ethics board. If you are unable to adhere to our open data policy, please kindly revise your statement to explain your reasoning and we will seek the editor's input on an exemption. Please be assured that, once you have provided your new statement, the assessment of your exemption will not hold up the peer review process.

**Reply:** We have completed the data availability statemen.

5.Please review your reference list to ensure that it is complete and correct. If you have cited papers that have been retracted, please include the rationale for doing so in the manuscript text, or remove these references and replace them with relevant current references. Any changes to the reference list should be mentioned in the rebuttal letter that accompanies your revised manuscript. If you need to cite a retracted article, indicate the article’s retracted status in the References list and also include a citation and full reference for the retraction notice.

**Reply:** All references in this manuscript are complete and correct, and no citations are made to papers that have been retracted. In this revision, a reference has been added to this manuscript, the specific reference is [26].

[26] Zhao, Z. Sample-based Dynamic Event-triggered Algorithm for Optimization Problem of Multi-agent Systems. International Journal of Control, Automation and Systems. 2022;20(8):2492–2502.

**Additional Editor Comments:** Two reviews are obtained for this submission. Both reviewers are positive about this work, but one of them suggests that it can be improved by providing more explanation on the implementation process of the proposed optimization algorithm and the meanings of the variables. The authors are encouraged to address these comments appropriately.

**Reply:** Thank you for the additional editor comments. We appreciate the feedback from both reviewers and their positive assessment of this work. We acknowledge these suggestions and will ensure that the comments are addressed in the revised manuscript.

**Response to Reviewer 1**

**Comments to the Author:** The manuscript provides a comprehensive study on the optimization problem of MAS under undirected graphs, which is a relevant and important research area. The use of a zero-gradient-sum (ZGS) algorithm with a dynamic event-triggered (DET) mechanism is novel and promising. It effectively reduces the reliance on continuous state information from neighbors, which can lead to improved efficiency in multi-agent systems. The following comments should be taken into account for further improvement.

**Reply:** Thank you for your accurate summary and positive comments. Next, we will proceed to explain the specific modifications made regarding the detailed reservations and suggestions you provided.

**Comment 1.** The paper proposes a method to optimize multi-agent systems under undirected graphs by reducing the communication frequency among agents. However, the description of the implementation process of the proposed optimization algorithm is not sufficiently thorough. It is recommended to provide a more detailed and specific description.

**Reply:** Thank you very much for your valuable feedback. We have incorporated your suggestions by providing a detailed description of the implementation process for the proposed optimization algorithm in the manuscript. In detail, we display the modified part below：

In this paper, we adopt a periodic sampling method to obtain the state of the agent, where the sampling period is defined as $h$. This means that we sample the state of the agent at intervals of $h$. Specifically, we use $x_{i}(t)$ to represent the state of the agent at time $t$, and this state changes dynamically over time. Firstly, the states of the neighbors and the agent's own state at the previous triggering time are sent to the controller to obtain the agent's current state. Simultaneously, an event detector is installed on each agent, which only checks if the triggering condition is met at the sampling time. Subsequently, the agent's state from the previous sampling time is sent to a constructed trigger for evaluation. When the preset triggering condition is satisfied, the agent's state at the triggering time is obtained. Finally, the obtained local state is used to update the control actions of both the agent itself and its neighbors. In practice, when the value at the sampling point exactly meets the DET condition, we have $x_{i}(t)=x_{i}(\varsigma h)=x_{i}(t_{k}^{i})$. Due to the existence of time delay in practical transmission, this section considers satisfying a delay of $\tau\in(0,h)$. In addition, for $t\in\left[\varsigma h+\tau,(\varsigma+1)h+\tau\right)$, $\varsigma=0,1,2,\ldots$, we define $\hat{x}_{i}(t)=x_{i}(t_{k}^{i})$ and deviation variable $\delta_{i}(t)=x_{i}(t)-\hat{x}_{i}(t)$. The main structure of the algorithm is designed as follows:

**Comment 2.** The meanings of the variables used in the formulas are not sufficiently explained, such as the specific reference of positive parameters related to agent . It is recommended that detailed explanations and definitions be provided in the manuscript.

**Reply:** Thank you for your valuable feedback. We sincerely apologize for our oversight in not adequately explaining the meanings of the variables in the manuscript. We have thoroughly reviewed and provided comprehensive explanations for the variables in the manuscript. For your convenience, the details are modified as follows:

where $d_{i}$ is the degree of the agent $i$, $\gamma$ is a positive constant. $\varepsilon_{i}$，$\mu_{i}$，$\omega_{i}$ are self-designable parameters. The deviation variable $\delta_{i}(lh)=x_{i}(lh)-\hat{x}_{i}(lh)$.

**Remark1:** Considering that it takes some time to make ET judgments on agent states. When designing the DET condition (8), we use the agent state$q_{i}(\varsigma h-h)$ at the previous sampling moment, which is more logical. In addition, the agent state is taken into account when designing DET conditions, which makes the frequency of triggering not increase when the agent state is close to the optimal value. In the design of DET conditions, different parameters $\varepsilon_{i}$，$\mu_{i}$，$\omega_{i}$ can be set for each agent according to different needs to ensure that it can make the most accurate response in a specific situation. For the convenience of analysis, this paper chooses to set the same $\varepsilon_{i}$，$\mu_{i}$，$\omega_{i}$ for each agent in order to study its behavior and response ability more clearly.

**Comment 3.** It is better to give a specific definition and explanation of "allowing the sampling period to be arbitrarily large".

**Reply:** We sincerely thank you for your thorough review of our paper and constructive comments. In this study, we have validated the aspect of " allowing the sampling period to be arbitrarily large " through both theoretical analysis and simulation experiments. Firstly, based on the stability conditions derived from Theorem 2, it can be observed that when the sampling period $h$ selects a small value, $\tau$ and $h$ can be eliminated due to $0<\tau<h$; When the sampling period $h$ takes a large value, the term can be ignored. In our simulation experiments, we found that selecting larger sampling periods requires more time steps, but eventually leads to optimal solutions. Conversely, in reference [26], excessively large sampling periods hinder the satisfaction of stability conditions, resulting in the algorithm's failure to converge and find the optimal solution. Hence, the innovation of our proposed algorithm lies in its ability to accommodate arbitrarily large sampling periods. In this study, the convergence of the algorithm is not affected by the sampling period, meaning that regardless of the chosen duration, the algorithm will ultimately converge to the optimal solution. However, selecting larger sampling periods can significantly reduce the communication frequency among agents, albeit at the cost of slower convergence speed. Therefore, while our study allows for the selection of arbitrary sampling periods, a trade-off between convergence speed and communication frequency needs to be made in order to determine the optimal sampling period. In the paper, we have added further discussion on this aspect. For your convenience, the modified section is as follows:

**Remark3:** The stability conditions derived from Theorem 2 reveal that when the sampling period $h$ is chosen to be small, $\tau$ and $h$ can be eliminated due to $0<\tau<h$; When the sampling period $h$ takes a large value, the term can be ignored. From the perspective of simulation experiments, longer sampling periods require more time steps, but still lead to optimal solutions. However, in [26], excessively large sampling periods make it difficult to satisfy the stability conditions, resulting in the algorithm's failure to converge and find the optimal solution. Therefore, the algorithm proposed in this paper allows the sampling period to be arbitrarily large, which is of great significance.

**Response to Reviewer 2**

**Comments to the Author:** This paper studied the optimization problem of multi-agent systems. The zero-gradient-sum (ZGS) algorithm based on dynamic event-triggered (DET) mechanism is investigated to reduce the communication frequency among agents. This paper can be accepted.

**Reply:** We appreciate your positive evaluation of the paper. We are glad to hear that this paper on the optimization problem of multi-agent systems meets your acceptance criteria.
